# Catechins-Modified Selenium-Doped Hydroxyapatite Nanomaterials for Improved Osteosarcoma Therapy Through Generation of Reactive Oxygen Species

**DOI:** 10.3389/fonc.2019.00499

**Published:** 2019-06-13

**Authors:** Suliman Khan, Muhammad Wajid Ullah, Rabeea Siddique, Yang Liu, Ismat Ullah, Mengzhou Xue, Guang Yang, Hongwei Hou

**Affiliations:** ^1^The Department of Cerebrovascular Diseases, The Second Affiliated Hospital of Zhengzhou University, Zhengzhou, China; ^2^The Key Laboratory of Aquatic Biodiversity and Conservation of Institute of Hydrobiology, Chinese Academy of Sciences, Wuhan, China; ^3^University of Chinese Academy of Sciences, Beijing, China; ^4^Department of Biomedical Engineering, Huazhong University of Science and Technology, Wuhan, China; ^5^Henan Medical Key Laboratory of Translational Cerebrovascular Diseases, Zhengzhou, China; ^6^State Key Laboratory of Materials Processing and Die/Mold Technology, School of Materials Science and Engineering, Huazhong University of Science and Technology, Wuhan, China

**Keywords:** osteosarcoma, catechins, selenium, hydroxyapatite, ROS, cancer therapy

## Abstract

Osteosarcoma is the most common bone cancer with limited therapeutic options. It can be treated by selenium-doped hydroxyapatite owing to its known antitumor potential. However, a high concentration of Se is toxic toward normal and stem cells whereas its low concentration cannot effectively remove cancer cells. Therefore, the current study was aimed to improve the anticancer activity of Se-HAp nanoparticles through catechins (CC) modification owing to their high cancer therapeutic value. The sequentially developed catechins modified Se-HAp nanocomposites (CC/Se-HAp) were characterized for various physico-chemical properties and antitumor activity. Structural analysis showed the synthesis of small rod-like single phase HAp nanoparticles (60 ± 15 nm), which effectively interacted with Se and catechins and formed agglomerated structures. TEM analysis showed the internalization and degradation of CC/Se-HAp nanomaterials within MNNG/HOS cells through a non-specific endocytosis process. Cell toxicity analysis showed that catechins modification improved the antitumor activity of Se-HAp nanocomposites by inducing apoptosis of human osteosarcoma MNNG/HOS cell lines, through generation of reactive oxygen species (ROS) which in turn activated the caspase-3 pathway, without significantly affecting the growth of human normal bone marrow stem cells (hBMSCs). qPCR and western blot analyses revealed that casp3, p53, and bax genes were significantly upregulated while cox-2 and PTK-2 were slightly downregulated as compared to control in CC/Se-HAp-treated MNNG/HOS cell lines. The current study of combining natural biomaterial (i.e., catechins) with Se and HAp, can prove to be an effective therapeutic approach for bone cancer therapy.

## Introduction

Cancer is the major health concerns and the second leading cause of death all over the world (1). Osteosarcoma, a commonly known bone cancer, cause malignant primary bone tumor with a high mortality rate, both in children and adolescents (2). It can easily metastasize to lungs after its germination in the distal femur and proximal tibia (3–8). In addition, the difficulty in cleaning up after the treatment increases the probability of recurrent osteosarcoma. Furthermore, the defects caused by surgery need to be filled for bone repair to relieve the physical limitations to patients. Therefore, extensive efforts have been devoted to the development of advanced targeted drug delivery systems and heat mediators to regulate oncogenes and tumorigenesis in treating osteosarcoma (9, 10).

The bone strength mainly relies on selenium (Se), calcium (Ca), and vitamins (K and D) contents. It is further enhanced by the addition of several other trace elements, such as manganese (Mn), zinc (Zn), fluorine (F), copper (Cu), magnesium (Mg), strontium (Sr), boron (B), and iron (Fe), etc. (11–15). Se deficiency is associated with the risk of developing multiple cancers; such as in bone, breast, ovary, prostate, gastrointestinal tract, and lungs (16, 17). To minimize the risk associated with the Se deficiency, its doping with hydroxyapatite (HAp) can be an effective approach which may potentially reduce the growth of osteosarcoma cells. Currently, HAp has received immense consideration in reconstructive surgeries, orthodontic, orthopedic substances, and three-dimensional printing of scaffolds owing to its high bioactive and osteoconductive properties (18–20). Its large surface area allows it to strongly interact with the neighboring bone and connective tissues *in vivo*. Se prevents the cancer development through generation of reactive oxygen species (ROS) (16); however, it possesses low anticancer activity when used at low concentrations, while its higher concentration can potentially inhibit the growth of normal cells leading toward osteosarcoma (21). This necessitates the improvement of its anticancer activity while still retaining its low or no toxicity toward the normal cells. To this end, its modification with another anticancer reagent to improve its antitumor activity can have the additive effect toward the osteosarcoma cells.

Green tea contains several important chemical reagents, among which 30% are catechins including epigallocatechin gallate (EGCG), epigallocatechin (EGC), epicatechin gallate (ECG), and epicatechin (EC). These play a preventive role against the development of different types of cancers (22–25). A recent study by Stadlbauer et al. suggested that epicatechin-3-O-gallate and 5,7-difluoro-epicatechin-3-O-gallate can potentially prevent the tumorigenesis during the initiation, promotion, and progression of cancer by diminishing the inflammation level through reduction of inflammatory lymphocytes (26). Similarly, EGCG affects several signal transduction pathways related to cancer development and exhibits strong anticancer activity by targeting several cell signaling pathways causing tumor growth suppression, induction of apoptosis through generation of reactive oxygen species, and inhibition of metastasis and angiogenesis (25, 27–30). In addition, it also exerts anticancer activity by acting as a chemo/radio-sensitizer when combined with conventional therapies (31). *In vitro* and *in vivo* studies have demonstrated that catechins control the cancer development by different mechanisms, such as through induction of apoptosis to control the cell growth arrest, through altered expression of cell-cycle regulatory proteins, by activating killer caspases, and through suppression of nuclear factor kappa-B activation (23). Catechins also act as carcinoma blockers by modulating the signal transduction pathways, involved in cell proliferation, transformation, inflammation, and metastasis (29, 32–37).

Owing to the known antitumor properties of catechins, the current study was aimed to develop catechins-modified Se-doped HAp nanocomposites (CC/Se-HAp) for potential application in osteosarcoma therapy. The developed nanocomposites were characterized by various physico-chemical and biological properties. The sequential self-assembly of green tea-derived catechins with Se-doped HAp resulted in formation of stable nanocomplexes which showed improved anticancer activity *in vitro* as compared to Se-doped HAp nanocomposite. These nanocomposites enhanced the ROS-mediated apoptosis through activation of caspase-3 pathway. These findings demonstrate the antitumor potential of the developed catechin-modified Se-doped HAp nanocomposites with the improved outcome to prevent the adverse and toxic effects of high concentration of Se toward the normal cells for cancer therapy.

## Materials and Methods

### Materials

The chemical reagents, including calcium nitrate tetrahydrate (Ca(NO_3_)_2_.4H_2_O) and sodium selenite (Na_2_SiO_3_), were purchased from National Medicine Chemical Reagent Company (China). Ammonium hydrogen phosphate ((NH_4_)_2_HPO_4_) was purchased from Regal Biotech Technology, Inc. (Shanghai, China), whereas sodium polyacrylate [[[Inline Image]]CH_2[[InlineImage]]_CH(CO_2_Na)[[Inline Image]]]_*n*_ (PPAS) (MW: 5100) from Sigma-Aldrich (St. Louis, MO, USA). Commercial Brazilian green tea was purchased from a tea center (Peshawar, Pakistan). In all experiments, ultrapure deionized (DI) distilled water was used. Dulbecco's Modified Eagle Medium (DMEM), fetal bovine serum (FBS), eagle's modified minimum essential medium (MEM), streptomycin, and penicillin were obtained from Hyclone (USA). The cell counting kit-8 (CCK-8) was purchased from Sigma Aldrich (St. Louis, USA).

### Cell Culturing

Human osteosarcoma cell lines (MNNG/HOS) were kindly provided by Tongji Medical College of Huazhong University of Science and Technology, Wuhan, China. The human bone marrow stem cells (hBMSCs) were purchased from Chinese Center of Type Culture Collection of Wuhan University, Wuhan, China. The MNNG/HOS and hBMSCs cells were cultured in MEM and DMEM media, respectively. Both culture media were supplemented with 10% fetal bovine serum, 100 mg/mL streptomycin, and 100 unit/mL penicillin, and kept in an incubator at 37°C (5% CO_2_, 95% relative humidity). The culture media for both cell lines were refreshed after 24 h.

### Synthesis of HAp Nanoparticles

The pristine HAp nanoparticles were synthesized *via* aqueous precipitation method followed by sonication technique using calcium nitrate tetrahydrate (Ca(NO_3_)_2_·4H_2_O), ammonium hydrogen phosphate ((NH_4_)_2_HPO_4_), and ammonium hydroxide (HN_4_OH) solution as reported previously (15). A schematic representation of preparation of HAp nanoparticles is shown in Supplementary Figure S1. Briefly, 1.0 M calcium nitrate tetrahydrate solution was prepared in DI water with the desired concentrations of sodium selenite. The pH was adjusted to 10.5 with 25% (v/v) ammonium hydroxide solution. Thereafter, 0.6 M ammonium hydrogen phosphate solution (pH ≥ 9) was added dropwise (2.0–2.5 mL/min) into the cationic mixture to form a white precipitated mixture. The precipitated mixture was stirred for 4 h at 70°C, using sodium polyacrylate (PPAS, MW 5100) as a dispersant, followed by sonication for 20 min and allowed to settle down at room temperature for 24 h, until the formation of gel. The gel was collected *via* centrifugation, washed three-times with DI water, and dried at 60°C in a hot air oven.

### Synthesis of Se-Doped HAp and CC/Se-HAp Nanocomposites

The stoichiometric Se-doped HAp and CC/Se-HAp nanocomposites were prepared by a modified aqueous co-precipitation method (16), using ammonium hydrogen phosphate, calcium nitrate tetrahydrate, and sodium selenite were used as sources of phosphorous (P), calcium (Ca), and selenium (Se), respectively (Table S1). Briefly, Se-HAp nanomaterial was prepared through dropwise and simultaneous addition of aqueous solutions of 5.45 mM (NH4)_2_HPO_4_ and 0.55 mM Na_2_SeO_3_ into the aqueous solution of Ca(NO_3_)_2_·4H_2_O, under vigorous stirring at 70°C. The pH was adjusted to 10.5 with 25% (v/v) ammonium hydroxide solution (Supplementary Figure S1, middle). The precipitate was stirred continuously for 24 h at 70°C, using PAAS as a dispersant, until the formation of a semitransparent and well-dispersed gel. This gel was then collected *via* centrifugation, washed three-times with DI water, and dried at 60°C in a hot air oven. For preparation of CC/Se-HAp nanocomposites, different catechins solutions (CC-1, CC-2, and CC-3) were prepared from green tea (Supplementary Material), and subsequently used to prepare CC/Se-HAp-1, CC/Se-HAp-2, and CC/Se-HAp-3 nanocomposites, respectively (Supplementary Figure S1).

### Characterization

The synthesized pristine HAp nanoparticles, and Se-HAp and CC/Se-HAp nanocomposites were characterized for various structural and chemical properties. The phase composition of synthesized HAp, Se-HAp, and CC/Se-HAp was investigated by using XRD (PANalytical B.V., Netherlands). The morphology of synthesized nanomaterials was examined by transmission electron microscopy (TEM, Tecnai G2 20, FEI, Holland), while structural analysis was carried out using Gemini scanning electron microscope, SEM 300 (Zeiss Germany). FTIR (Vertex 70, Bruker, German) analysis was carried out to investigate the functional groups present in the pristine nanoparticles, using the classical KBr pellet system technique in transmission mode (See Supplementary Material for detailed information).

### Cellular Uptake Analysis

The cellular uptake of CC/Se-HAp nanomaterials was determined through TEM analysis (38). Briefly, the synthesized nanomaterials were directly added to the culture dishes, separately containing human osteosarcoma cell line (MNNG/HOS), at a concentration of 50 μg/mL and cultured in MEM medium for 12 h. The medium was changed after every 2 days. For TEM analysis, the cell sections were immediately prepared and observed with a specific cell TEM (H-7000FA, HITACHI, Japan) by following a previously published protocol (39). Briefly, the overnight cultured seeded cells (1 × 10^6^ cells per well) were treated with nanomaterials in reduced serum (MEM) and cell monolayers were rinsed with D-PBS and fixed in a mixture of 2% paraformaldehyde, 2.5% glutaraldehyde, and 0.15 M sodium phosphate at pH 7.4, and incubated overnight at 37°C. The monolayers were fixed in a mixture of 1% osmium tetroxide, 1.25% potassium ferrocyanide, and 0.15 M sodium phosphate buffer and rinsed in DI water. Cells were embedded in polybed epoxy resin after dehydrating them using acetone. Finally, the ultrathin sections were stained with 4% aqueous uranyl acetate and Reynolds' lead citrate and observed under TEM.

### *In vitro* Cytotoxicity Assay

The cytotoxic effects of Se-HAp, CC/Se-HAp, and NaSeO_3_ (control) against human bone marrow stem cells (hBMSCs) and human osteosarcoma cell line (MNNG/HOS) were measured using a CCK-8 assay kit. Briefly, the cells were seeded at a density of 1 × 10^4^ cells per well in a 96-well plate followed by adding 100 μg/mL of each nanomaterial to respective well. The plates were incubated for 6, 12, 18, 24, 36, 42, and 48 h. After incubation, the portion of viable cells was determined using CCK-8 assay according to the manufacturer's protocol (Supplementary Material). Optical density (OD) values were measured for all samples at 450 nm using a microplate reader (Eon, BioTek, USA). The viability of cells was expressed as a percentage of untreated control cells.

### Caspase-3 Activity Assay

Caspase-3 activity was assessed calorimetrically using the CaspACE Assay System (Promega, Madison, WI, USA), following the manufacturer's instructions. The MNNG/HOS cells at a density of 1 × 10^6^ cells per well were treated with caspase inhibitor Ac-DEVD-CHO (or antioxidant N-acetyl-cysteine). Cells were then treated with CC/Se-HAp nanomaterials and incubated for 18 h prior to lysis. Cell lysates were incubated with the caspase-3 substrate for 4 h. Free Ac-DEVD-p-nitroaniline was monitored by a spectrophotometer at 405 nm.

### Determination of Intracellular ROS Level

ROS generation was measured by a previously reported method using a non-fluorescent probe, 2,7-diacetyl dichlorofluorescein (H_2_DCFDA) (16, 40). Briefly, the MNNG/HOS cells were cultured in 6 and 24-well plates at a density of 1 × 10^6^ cells per well, for 16 h and incubated with CC/Se-HAp (100 μg/mL), HAp (100 μg/mL), and sodium selenite (2 μM) for 6, 8, 10, and 24 h. The cells were then incubated with 1 μM of DFCH/DA for 30 min at 37°C and washed three times with phosphate buffer saline (PBS) and resuspended in PBS. ROS generation was measured by flow cytometry (Cytomics FC500, Beckman Coulter, U.S.A.) at 485 nm excitation and 538 nm emission wavelengths. To investigate the inhibition effect of ROS generation in the presence of CC/Se-Hap, N-acetylcysteine (NAC) was added to each well and cultured for 1 h before incubation with materials under investigation. The inhibition effect of ROS generation was also confirmed by adding MnTMPyP (10 μM) to each well and cultured for 1 h before incubation with the nanomaterials.

### Western Blot Analysis

Cells were cultured and treated with Na_2_SeO_3_, Se-HAp, and CC/Se-HAp. The cells were then lysed using lysis buffer on ice for 20 min and centrifuged at 12,500 rpm for 10 min at 4°C. The protein concentration was determined by BCA assay (Thermo-Fisher Scientific) according to the manufacturer's instructions. Loading of 60 μg protein onto SDS-polyacrylamide gel was followed by electrophoresis and transferred to polyvinylidene fluoride (PVDF) membrane. The membrane was blocked with blocking buffer containing TBS [Tween-20 (0.1%), tri-sec-buffer saline (10%), DI water (89.9%)], and 5% skimmed milk for 2 h and incubated overnight at 4°C with monoclonal antibodies against gapdh, p53, bcl-2, caspase3, caspase9, cox-2, nf-kb, and bax. The membranes were then soaked with horseradish peroxidase (HPR)-conjugated secondary antibodies for 1 h at room temperature. The immunoblots were observed using chemiluminescence system (Bio-Rad, Hungary Ltd) according to the manufacturer's instructions.

### Apoptosis Analysis

The qualitative apoptosis of MNNG/HOS cells was determined by treating comparable number of cells (1 × 10^6^ cells per well) with both Na_2_SeO_3_ and CC/Se-HAp nanomaterials in a 12-well plate. The cells were washed with PBS and fixed in cold methanol: acetone solution for 5 min. The cells were then treated with DAPI (4 μg/ml 4,6-diamidine-2-phenylindole dihydrochloride) at room temperature for 10 min. The cells were examined by fluorescence microscopy (Olympus) at 200 × magnification. For quantitative apoptosis study, MNNG/HOS cells were seeded onto 12-well plates (10^6^ cells/well). After incubation, the cells were treated with Na_2_SeO_3_ and CC/Se-HAp, keeping untreated cells as a control. The cells were trypsinized after incubation for 24 h, collected, and resuspended in 350 μL binding buffer. Then 4 μL annexin V-FITC and 8 μL PI were added to the cell suspension and mixed in dark for 15 min prior to flow cytometry analysis.

### Quantitative Real-Time Polymerase Chain Reaction

The apoptosis-related gene expression levels were analyzed using real-time PCR. The total RNA of cells, both treated with the nanomaterials under investigation and control, was extracted by using Trizol reagent according to the manufacturer's instructions (Thermo-Fisher, Applied Biosystems). The RNA concentration was measured using a spectrophotometer. cDNA was synthesized using a high capacity RNA to cDNA kit (Thermo-Fisher, Applied Biosystems). qPCR analysis was performed according to the standard protocol (Applied Biosystems). The forward and reverse primers were generated through Primer-BLAST (NCBI) and/or primer-3.0 (Table S2). Relative gene expression was quantified by the ΔΔCT method using ACTIN as a reference gene.

### Statistical Analysis

Results were expressed as means ± SD for each group. The analyses were performed using the two-tailed student's *t*-test. Statistically significant differences between the control and CC/Se-HAp nanomaterials were considered at ^*^p < 0.05. All statistical analyses were performed in Prism Software (Graph Pad Prism 7, La Jolla, CA, USA) and MS Excel 2016 (Microsoft).

## Results

### Synthesis of CC/Se-HAp Nanomaterials

The catechins-modified selenium-doped hydroxyapatite nanoparticles (CC/Se-HAp) were synthesized through the aqueous precipitation method (Figure S1). According to the previous reports, selenium replaces phosphate during the synthesis of HAp nanoparticles (16, 19, 21, 41). The synthesized Se-HAp nanoparticles were modified with catechin contents through vigorous mixing in a sonicator. It was hypothesized that catechins modification of Se-HAp nanoparticle will enhance their anticancer activity owing to the known anticancer activities of individual catechins and Se-HAp nanoparticles. The prepared nanomaterials were characterized for various physico-chemical properties such as size, chemical structure, structural morphology, and colloidal stability, which are important factors in the designing of nanoparticles (39).

### Structural Analysis of CC/Se-HAp Nanomaterials

**Figure 1:**
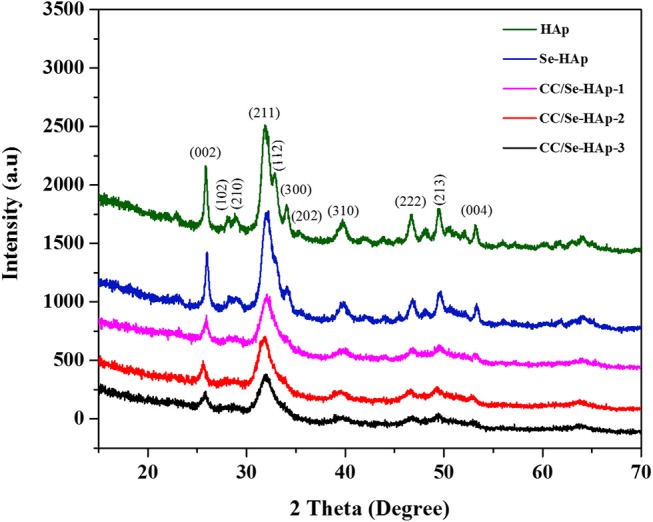
X-ray diffraction spectra of pristine HAp nanoparticles, and selenium-doped hydroxyapatite (Se-HAp), and modification of Se-HAp composites with different concentrations of catechins, i.e., 5% (CC/Se-HAp1), 7% (CC/Se-HAp2), and 10% (CC/Se-HAp3). The patterns of XRD depicted that both HAp and Se-HAp presented the typical HAp peaks, with the no significant differences. The peak (211) associated CC/Se-HAp showed a reduction in crystallinity which determines the association of catechin contents.

XRD analysis of the as-prepared nanomaterials was carried out to investigate the basic polymorphic structure of HAp and any structural variations during the Se-doping and catechins modification (Figure 1). The XRD spectrum of the extended linear scanning (10–70°) of HAp showed characteristic peaks confirming its successful synthesis, where the peaks were comparable with the standard HAp (JCPDS card no. 24-0033). Further, the intense and sharp peaks indicate the crystalline nature of synthesized HAp nanoparticles. The Se-doping of HAp slightly altered its crystallinity as indicated by the less intense crystallinity peaks. The Se-HAp and CC/Se-HAp samples showed a single phase (solid solution) with a hexagonal structure [space group, p63/m (176)]. The XRD spectra of CC/Se-HAp-1, CC/Se-HAp-2, and CC/Se-HAp-3 further indicated the synthesis of crystalline nanoparticles with crystallinity scope inferior to pristine HAp and Se-HAp. This crystallinity scope was further decreased with the increased concentration of catechins. Additionally, the peak intensities were slightly shifted toward higher 2θ value and three major differential peaks (211), (112), and (300) were merged into single peak, which indirectly indicates the substitution effect with nanosized features. The effect of Se-doping and catechins modification on lattice parameters (a and c), unit cell volume (Å)^3^, crystal size (D), and crystallinity (X_c_) are summarized in Table 1.

**Table 1:** Summary of effect of selenium and catechins contents on the lattice parameters (a and c), unit cell volume, crystal size, and crystallinity of pristine HAp and catechins-modified selenium-doped HAp nanocomposites.

**Sample**	**Lattice parameters (Å)**		**Unit cell volume (Å)^**3**^**	**Crystallinity (X_**c**_)**	**Crystal size (D_**002**_) nm**
	**a**	**c**			
HAp	9.4482	6.883	532.10	1.612	29.78
Se-HAp	9.3804	6.8547	522.33	1.13	25.45
CC/Se-HAp1	9.3546	6.8499	519.18	0.22	21.58
CC/Se-HAp2	9.3215	6.8676	516.76	0.20	18.91
CC/Se-HAp3	9.2989	6.8442	512.51	0.13	15.89

### Chemical Structure Analysis of CC/Se-HAp Nanomaterials

**Figure 2:**
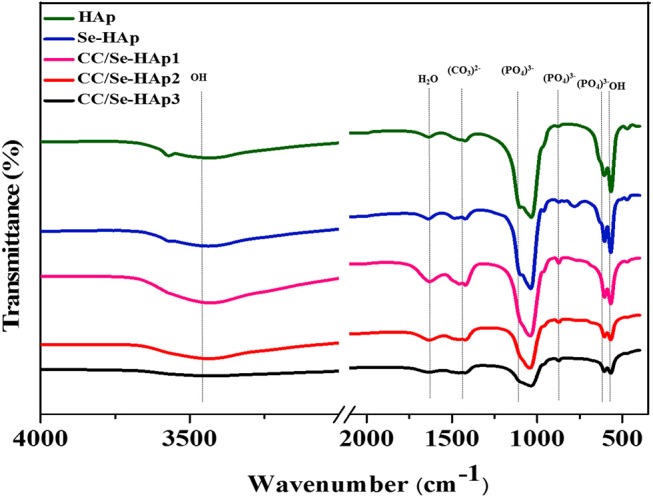
Fourier transform infrared spectra of pristine HAp, Se-HAp, and its modification with different concentrations of catechins, i.e., 5% (CC/Se-HAp1), 7% (CC/Se-HAp2), and 10% (CC/Se-HAp3). The peaks show that both HAp and Se-HAp presented the typical HAp functional groups, with a decrease in intensity (1,000 cm^−1^) upon catechins modification.

FTIR spectroscopy was used to investigate the presence of specific functional groups (Figure 2) (42). The peaks for O-H vibrations were observed at 633 cm^−1^ and 3,500–3,000 cm^−1^. The peak at 1,640 cm^−1^ was assigned to the bending mode of H_2_O molecule present in the HAp lattice, which are in agreement with previous reports (41, 43). The peaks observed at 1,486, 1,459, 1,420, 1,424, and 872 cm^−1^ correspond to the carbonate (C${\text{O}}_{3}^{2-}$) groups, which indicated its substitution at P${\text{O}}_{4}^{-3}$ (B-site) and OH positions (A-site) in the HAp lattice. The substitution of C${\text{O}}_{3}^{2-}$ in the OH group might result in an increased length of the unit cell toward the a-axis and a decline toward c-axis, as reported previously (44). Similarly, the peaks for stretching vibrations due to OH group were present at 3,570 cm^−1^ and 630 cm^−1^ (45, 46) in all spectra of HAp, Se-HAp, and CC/Se-HAp samples while its intensity was relatively low in the spectrum for Se-HAp nanocomposite.

### Structural Morphology of CC/Se-HAp Nanomaterials

**Figure 3:**
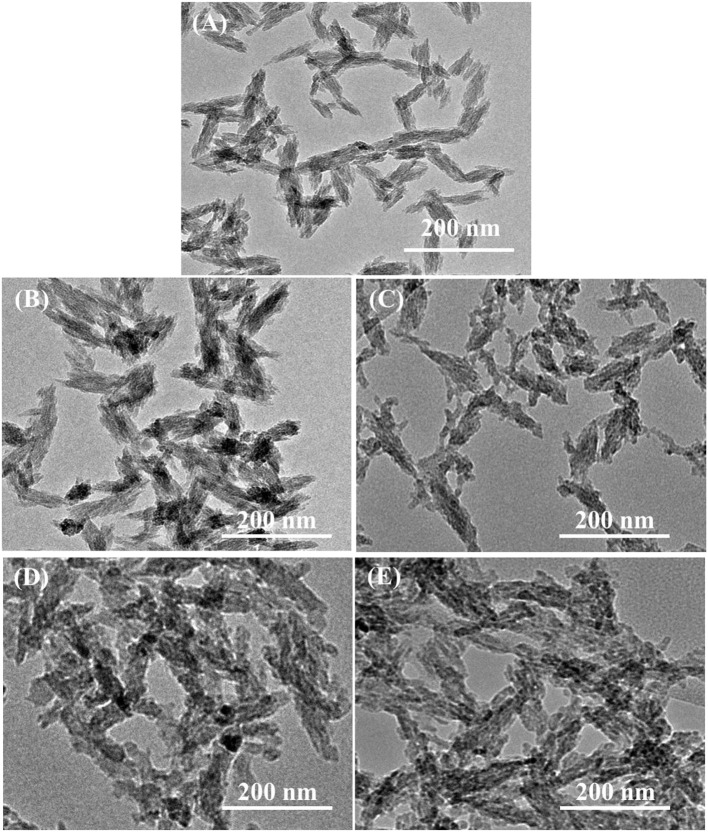
Transmission electron micrographs of **(A)** pristine HAp nanoparticles and **(B)** Se-HAp, **(C)** CC/Se-HAp1, **(D)** CC/Se-HAp2, and **(E)** CC/Se-HAp3 nanocomposites.

From Figure 3, TEM analysis showed the formation of small rod-like structure of various HAp samples under investigation. The modified aqueous precipitation method effectively maintained the rod-like morphology of HAp upon doping with Se and catechins modification, and were well-dispersed in PBS and formed a stable colloidal suspension even when stored for over a month.

**Figure 4:**
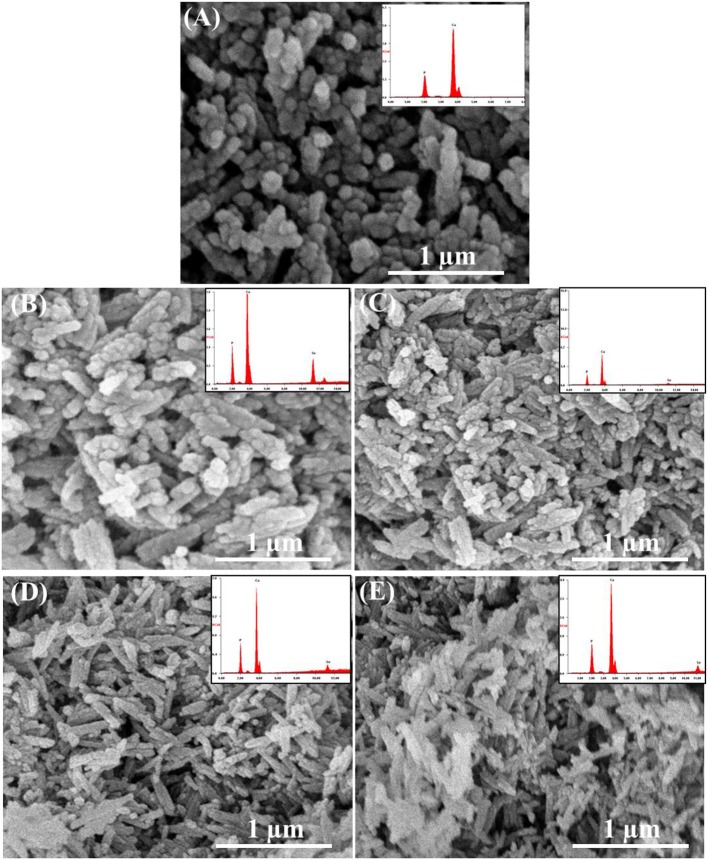
Field-emission scanning electron micrographs and XRF spectra (inset) of **(A)** pristine HAp nanoparticles, and **(B)** Se-HAp, **(C)** CC/Se-HAp1, **(D)** CC/Se-HAp2, and **(E)** CC/Se-HAp3 nanocomposites.

According to SEM micrographs (Figures 4A–E), the size of different HAp samples was estimated to be 60 ± 15 nm as determined *via* “Fiji v2” software, indicating their nano-crystalline features. The micrographs of pristine HAp and Se-HAp showed that the nanoparticles were nearly uniformly distributed and formed a moderately rough surface with slight pores, and particles were observed with dominant small rod-like morphology (Figure 4). In contrast, the Se-HAp samples with catechins content were highly agglomerated and exhibited dense rough surface with small rod-like morphology (Figures 4B–E). These results indicate that incorporation of Se and catechins content into HAp structure resulted in the synthesis of highly-agglomerated material and the particles exhibited small rod-like morphology. The strong agglomeration among the particles was due to the presence of catechins content which could be defined by the decreased particle size as confirmed *via* XRD analysis (Figure 1). XRF analysis was carried out to confirm the formation of HAp, Se-HAp, and CC/Se-HAp nanomaterials and to measure the degree of atomic percentage of Se substituted/doped during the synthesis of nanocomposites. The elemental mapping *via* XRF showed that Se distribution in CC/Se-HAp was reduced in comparison with HAp and Se-HAp where it was evenly distributed in the entire nanocomposite and was comparable to Ca and phosphate ions (Figures 3, 4). Overall, these findings imply that the incorporation of Se and catechins into HAp lattices did not alter the primary features of HAp nanoparticles including the physical dimension, geometrical shape, and crystal lattice. The integrated Se have the potential to be delivered *via* a degradation-mediated sustained release.

### *In vitro* Cytotoxicity of CC/Se-HAp Nanomaterials

**Figure 5:**
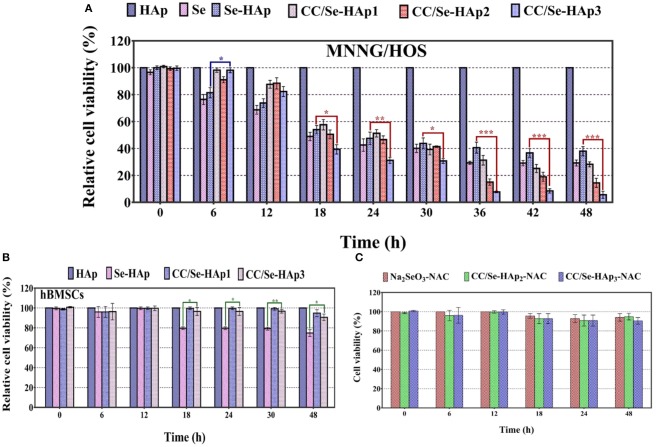
**(A)** Cytotoxicity analysis of human osteosarcoma cell line (MNNG/HOS) against Se-HAp and its modification with different concentrations of catechins, i.e., 5% (CC/Se-HAp1), 7% (CC/Se-HAp2), and 10% (CC/Se-HAp3) relative to control. **(B)** Viability analysis of human bone marrow stem cells (hBMSCs) on Se-HAp and its modification with 5% catechins (i.e., CC/Se-HAp1) and 10% catechins (i.e., CC/Se-HAp3) relative to control. **(C)** ROS inhibitor attenuated the cytotoxic effect of nanoparticles, which resulted in higher cell viability. ^*^p < 0.01, ^**^p < 0.001, ^***^p < 0.0001 indicate significant differences of CC/Se-HAp-3 in comparison with Se-HAp.

The *in vitro* anticancer activity of CC/Se-HAp nanomaterials was investigated against MNNG/HOS cells, using CCK-8 assay kit. The principle of this assay is that the color of developing substrate WST-8 is reduced by the intracellular dehydrogenase in live cells to water-soluble orange colored formazan, which is directly quantified photo-metrically and its absorbance reflects the number of viable cells in the culture medium (47). As shown in Figure 5A, the Se-HAp is much less toxic to MNNG/HOS cells as compared to CC/Se-HAp, confirming the improved activity of CC/Se-HAp against MNNG/HOS cells upon catechins modification. Further, CC/Se-HAp-3 showed high toxicity toward the cells as compared to CC/Se-HAp-1 and CC/Se-HAp-2 nanocomposites. In contrast, the Se-HAp nanoparticles showed toxicity toward MNNG/HOS; however, it was much lower than CC/Se-HAp nanocomposites. To investigate the safety of the developed CC/Se-HAp nanocomposites toward the normal cells, their toxicity effect was determined toward the stem cells (hBMSCs) and the results are shown in Figure 5B. The results showed significantly higher toxicity of Se-HAp toward hBMSCs as compared to CC/Se-HAp. Furthermore, the response of CC/Se-HAp to MNNG/HOS and its degradation behavior was also confirmed by cytotoxicity evaluation. It indicated that incubation time was directly associated with the cytotoxicity of nanoparticles (Figure 5C). For instance, the cell viability was mainly associated with the degradation of nanoparticles. Cell viabilities of CC/Se-HAp-3, CC/Se-HAp-2, CC/Se-HAp-1, Se-HAp, Se, and HAp were 98, 91, 98, 81, 76, and 100%, respectively, after the interaction of cells and nanoparticle for 6 h. Moreover, these viability values were 5, 14, 28, 37, 29, and 100%, respectively, after cell-nanoparticles interaction for 48 h. Se (representation for Na_2_SeO_3_) was used as a positive inhibition control, which showed prominent inhibition effect. Surprisingly, the inhibition effect of Se-HAp was superior to CC/Se-HAp up to 18 h; however, the cytotoxicity of CC/Se-HAp nanoparticles increased continuously until the maximum incubation period of 48 h. These results indicate that catechins and selenium contents of CC/Se-HAp affect the cell viability and lysosomal permeability. Higher catechin contents along with selenium in CC/Se-HAp nanomaterials result in lower cell viability due to faster degradation rate. The apoptosis during earlier incubation (0–24 h) might be attributed to the phase of burst release, whereas the high cytotoxic effect could be due to the sustained release during the extended incubation (24–48 h). These results demonstrate that catechins modification of Se-HAp increased its toxicity toward the cancer cell lines (MNNG/HOS) due to their degradation within the cell.

### Internalization and ROS Generation

**Figure 6:**
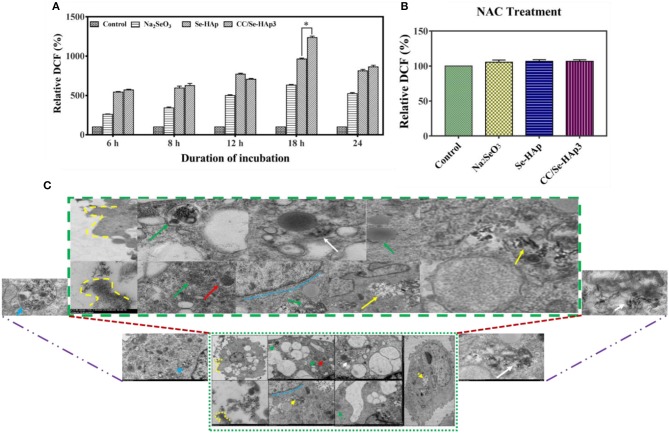
ROS generation and internalization of nanoparticles. **(A)** Relative fluorescence intensity of DCF (percent) generated during the process of ROS formation demonstrated that CC/Se-HAp exhibited a remarkable increase in DCF, as compared to Se-HAp or Na_2_SeO_3_. **(B)** Relative fluorescence intensity of DCF (percent) in presence of ROS inhibitor was non-detectable in presence of both catechin and selenium. **(C)** TEM micrographs illustrating the internalization of CC/Se-HAp nanomaterials into the human osteosarcoma cell through a typical endocytosis process. TEM images show that the nanoparticles were internalized by human osteosarcoma cells through a typical endocytosis process. Yellow dotted lines describe cell membrane invagination that is followed by endosome formation. Red arrows specifying region indicates newly generated endosomes loaded with nanoparticles. Green arrows determine the primary lysosome combined with the endosome. White arrows denote the degradation and release of active agents. Blue arrows represent the noticeable degradation of nanoparticles within the lysosome (secondary). Yellow arrows represent the nanoparticles in the vicinity of the nucleus (probably lysosomal disruption). ^*^p < 0.01 indicates significant differences of ROS generation in response to CC/Se-HAp treatment in comparison with Se-HAp treatment.

TEM micrographs indicated the internalization of CC/Se-HAp nanomaterials into MNNG/HOS cells *via* endocytosis. After encapsulated in the endosomes, the nanomaterials were transported to lysosome for intracellular degradation (Figure 6C, Figure S3) and increasing lysosomal permeability. The gradual degradation of internalized nanomaterials in the lysosomes was confirmed by their gradually increased activity with the incubation time. The anticancer drugs have been extensively reported to kill the cancer cells through ROS generation (16, 25, 48). Therefore, we investigated the underlying mechanism of CC/Se-HAp mediated apoptosis of cancer cells through ROS generation by hypothesizing that CC/Se-HAp nanomaterials can induce ROS production in mitochondria. To verify the proposed hypothesis, we detected the ROS generation at specific time intervals (0, 6, 8, 12, 18, and 24 h) by monitoring the “fluorescent product of DCF.” This unique ROS indicator was generated during the processes of ROS production (Figure 6A). The results showed the highest level of DCF in response to the treatment of cancer cells with CC/Se-HAp-3. This analysis further indicated that CC/Se-HAp-3 nanoparticles induced highest ROS production after cell-nanomaterials interaction for 18 h. This ROS produced level was significantly higher than that of Se-HAp and Se (Na_2_SeO_3_). These results depicted that such a low cell viability caused by CC/Se-HAp was induced by ROS generation that caused cell apoptosis. To further confirm these results, we inhibited the ROS generation by treating the cancer cells with *N-acetylcysteine* (NAC) prior to nanoparticles treatment. With this treatment, the CC/Se-HAp-3 treatment indicated almost 100% cell viability of cancer cells, as the nanoparticles could not induce the apoptosis after the inhibition of intracellular ROS generation (Figure 6B). The inhibition of ROS generation also inhibited the cytotoxic effect of nanoparticles (Figure 6C). Therefore, these results depicted that ROS generation is a key player in the process of CC/Se-HAp mediated cancer cells apoptosis.

### Regulation of Apoptosis-Associated Genes

**Figure 7:**
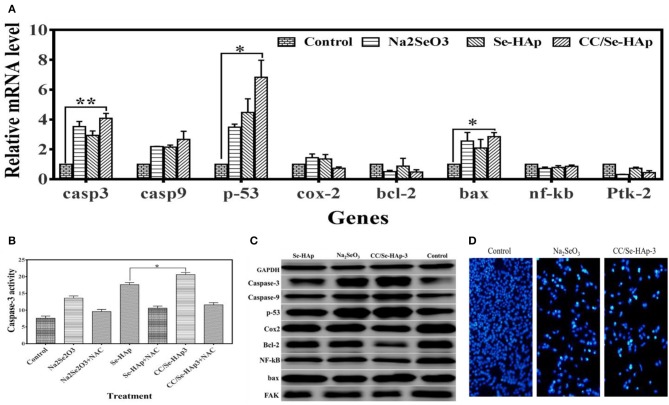
Molecular analysis associated with apoptosis of MNNG/HOS cells treated with nanoparticles. **(A)** Expression levels of genes (analyzed through qPCR) associated with cancer and/or apoptosis were altered, which indicate their association with nanoparticles treatment. **(B)** The caspase-3 activity assay was evaluated by using the CaspACE Assay System. The increase in caspase-3 determines that CC/Se-HAp-mediated apoptosis is involved caspase-3 pathway. **(C)** Key proteins involved in apoptosis and cancer were analyzed using western blot in order to confirm the qPCR results. Western blot indicates the alteration in protein levels in response to treatment of nanoparticles. **(D)** The qualitative analysis of apoptosis using fluorescence cytometry confirms the effectiveness of nanoparticles. ^*^p < 0.05 and ^**^p < 0.01 indicate significant differences in comparison with control.

The expression ratios of CASP-3, CASP-9, TP-53, COX-2, BCL-2, BAX, NF-kB, and FAK (PTK-2) in MNNG/HOS cells treated with nanomaterials for 24 h in comparison with non-treated cells, using qPCR are shown in Figure 7A. These findings demonstrate that the relative expressions of P-53, CASP-3, and BAX genes were significantly upregulated, while that of BCL-2 and COX-2 were slightly reduced. To further confirm the qPCR results of the ROS and apoptosis-related genes, their expression levels were measured using western blot technique. According to the western blot results, bcl-2, cox-2, and fak were down-regulated whereas caspase-3, caspase-9, p-53, nf-kb, and bax were upregulated in osteosarcoma cells in response to CC/Se-HAp nanoparticles treatment as compared to control (Figure 7C). These results are consistent with the qPCR results, which in turn are consistent with ROS generation and apoptosis.

It is well-documented that ROS generation induces caspase activation (17). Therefore, activation of caspase-3 was confirmed using CaspACE Assay System. The results showed that CC/Se-HAp induced caspase-3 activation in osteosarcoma cells (Figure 7B). The quantitative analysis showed that CC/Se-HAp activated higher levels of caspase-3 as compared to Se-HAp. These results strengthen the ROS-mediated apoptosis in CC/Se-HAP treated cancer cells. To further confirm the caspase-3 activation, the CC/Se-HAp-induced caspase-3 activity was determined by treating the osteosarcoma cells with caspase inhibitor using NAC. This treatment indicated similar caspase-3 levels in cancer cells treated with CC/Se-HAp-3, Se-HAp, and control. To further analyze the apoptotic effect of CC/Se-HAp nanoparticles, the osteosarcoma cells were treated with nanomaterials and analyzed through flow DAPI staining. The results demonstrated that nuclei of cells treated with nanomaterials were smaller and brighter stained with the crescent shape as compared to that of control cells which possessed intact and rounded nuclei with diffuse DAPI staining (Figure 7D). Furthermore, the cells showed separate globular structures (apoptotic bodies) around the periphery. The DAPI staining showed a dramatic increase in the number of apoptotic cells treated with CC/Se-HAp nanoparticles. DAPI staining revealed that exposure of cells to nanoparticles markedly increased the apoptosis, coincidently with the cytotoxicity test. In contrast, annexin V-FITC staining showed the notable percentage of early and late apoptotic cells in response to CC/Se-HAp treatment as compared to cells treated with Na_2_SeO_3_ (Figures S2, S3). These results demonstrate that CC/-Se-HAp nanomaterials enhanced the cellular apoptosis. Overall, these results indicate that two agents (i.e., catechins and selenium) simultaneously induced ROS generation for subsequent activation of caspase-3 and upregulation of P53, which led to higher apoptosis of cancer cells.

## Discussion

Natural bone is a hierarchically structured material assembled from basic building blocks of mineral nanoparticles in the frame of bone tissues and collagen protein, which become weak as a result of osteosarcoma (49). To treat osteosarcoma, different strategies including tissues cleaning and bone surgery are currently in practice; however, such strategies are not as accurate and lead to physical disability and recurrence of osteosarcoma. Therefore, targeted and specific therapeutic agents are required to completely kill the cancer cells. Hydroxyapatite is the main inorganic mineral in vertebrate bones and tooth enamels that has been extensively studied as excellent bone substitutes. Its doping with selenium has been reported previously as a potential anticancer agent (16, 17); however, it is toxic toward the normal cells when used at higher concentrations. Therefore, modification of Se-HAp nanoparticles with other anticancer agents, such as catechins, could be used to protect bone cancer metastasis and potentiate cancer cells removal. In the current study, a series of catechins-modified Se-doped HAp (CC/Se-HAp) nanocomposites were prepared and characterized for various physico-chemical and biological properties. In addition, the nanomaterials were evaluated for their size, morphology, and colloidal stability which are important factors in the designing of nanoparticles (39). Further, the developed nanomaterials showed a higher anticancer activity as compared to Se-HAp nanoparticles *in vitro*.

As reported previously, the nanoparticles having a size <100 nm possess better cell endocytosis (50–53) and mechanical compatibility with natural bone can effectively promote osteogenesis (54, 55). To this end, the developed nanoscale HAp nanoparticles (60 ± 15) can serve as ideal template for the development of new bone and effective nanocarrier in anticancer drug delivery system. TEM micrographs (Figure 3), showed that the developed nanomaterials possessed nano-dimensional size and single crystalline structure. Moreover, the CC/Se-HAp nanomaterials existed in a uniaxial-like form, i.e., larger dimensions in length (axial) and smaller dimensions in width (equatorial). Further, FTIR spectrum of pristine HAp showed the distinctive peaks for the triply degenerate υ_3_ asymmetric and non-degenerate υ_1_ symmetric of P-O stretching vibrations were present between 1,200 and 900 cm^−1^, which are in agreement with a previous report (46). Similarly, the double degenerate υ_2_ and triple degenerate υ_4_ bending of O-P-O were present between 600 and 400 cm^−1^ for all samples which is in accordance with the typical FTIR pattern of HAp (15, 41, 56).

Both selenium and catechin doses separately have been reported to have direct cytotoxic effects on malignant tumors *in vitro* and *in vivo* (16, 29, 31, 48). However, these particles are far behind the clinical studies due to their toxicity toward the normal cells and show low activity against cancer cells. Therefore, combining catechins with Se-HAp is expected to minimize the toxic effects of selenium toward the normal cells. Our data showed that CC/Se-HAp nanoparticles caused tumor growth inhibition through ROS generation in P53 mediated pathway, as indicated by the higher ROS generation and increased P53 levels (Figures 7A,D). Previous *in vitro* studies showed that Se-HAp nanoparticles induce ROS generation that leads to apoptosis (16). Comparatively, our data showed that CC/Se-HAp can induce higher cancer cell apoptosis (Figure 7A). In addition, the cytotoxicity results of CC/Se-HAp nanomaterials are comparable with a study which showed that nanoparticles with a size <100 nm possess better cell endocytosis efficiency as compared to those with a larger size (16). Therefore, the needle-like HAp nanoparticles prepared in the present study were expected to induce cytotoxicity and can effectively promote osteogenesis, as reported previously (16).

Generally, the cell viability is associated with the degradation of nanoparticles; therefore, the cell apoptosis was sufficiently higher after the cell-nanoparticles interactions for 24 h. These findings imply that Se and catechin contents in CC/Se-HAp nanoparticles after degradation in lysosome led to cell apoptosis. Researchers have explored the antitumor potential of different nanoparticles through ROS induction. For instance, Zn^2+^ could trigger ROS generation, thereby activates a p53-mediated apoptotic pathway (16, 57). Furthermore, catechins selectively induce ROS generation only in cancer cells (25). In current study, the higher levels of P53 and caspase-3 indicate the antitumor activities of selenium ions and catechin contents due to ROS generation. These findings are consistent with the earlier studies which showed that ROS generation could induce cancer cells apoptosis in P53 dependent pathway with the involvement of caspase-3 (16, 25). Studies have also shown that P53 activation in cancer cells induces the expression of P53 target genes which further leads to cell growth inhibition and apoptosis. Catechins also induce the activation of P53 in cancer cells (25, 58). Therefore, it is anticipated that CC/Se-HAp could affect the P53 mediated apoptosis pathway in a similar fashion. Furthermore, the internalization of nanoparticles on the surface is associated with the actin rearrangement near the plasma membrane and extension into the extracellular space (38). Similarly, the TEM micrographs (Figure 6C) revealed that most of the nanoparticles were rapidly internalized into the MNNG/HOS cells. This observation confirms the internalization and translocation of CC/Se-HAp nanoparticles. These observations are also in agreement with a previous report (16). Finally, the degradation of internalized CC/Se-HAp nanomaterials restrains the viability of cells as indicated by the reduced cell viability (Figure 6A). Furthermore, the internalization indicates that the nanomaterials were gradually degraded within the lysosomes with the increased incubation time, which is in agreement with a previously reported study of Se-doped HAp nanoparticles (16).

Overall, the results of present study indicate that CC/Se-HAp nanoparticles have the greater potency for the targeted treatment of the osteosarcoma with least side effects toward the normal stem cells. However, further analysis like release mechanism, release kinetics, action mechanism, and fate of the released particles from nanomaterials at different pH and with different concentrations of catechins and selenium, would provide the base for clinical trials of the developed therapeutic drug in the osteosarcoma therapy.

## Author Contributions

All authors contributed directly in the designing, experimentation, and write up of manuscript under the supervision of MX, GY, and HH.

### Conflict of Interest Statement

The authors declare that the research was conducted in the absence of any commercial or financial relationships that could be construed as a potential conflict of interest.

## References

[1] Siegel RL Miller KD Jemal A Cancer statistics, 2017. CA Cancer J Clin. (2017) 67:7–30. 10.3322/caac.21387 28055103

[2] Hao S Shen Y Wu H Meng J Xie L Wen T Modulatory effects of the composition and structure on the osteogenic enhancement for superparamagnetic scaffolds. Eng Sci. (2018) 4:100–10. 10.30919/es8d782

[3] Khanna C Wan X Bose S Cassaday R Olomu O Mendoza A. The membrane-cytoskeleton linker ezrin is necessary for osteosarcoma metastasis. Nat Med. (2004) 10:182–6. 10.1038/nm982 14704791

[4] Koshkina NV Kleinerman ES Waldrep C Jia S-F Worth LL Gilbert BE. 9-Nitrocamptothecin liposome aerosol treatment of melanoma and osteosarcoma lung metastases in mice. Clin Cancer Res. (2000) 6:2876–80. 10.1111/j.1749-6632.2000.tb07033.x 10914737

[5] Luu HH Kang Q Park JK Si W Luo Q Jiang W. An orthotopic model of human osteosarcoma growth and spontaneous pulmonary metastasis. Clin Exp Metastasis. (2005) 22:319–29. 10.1007/s10585-005-0365-9 16170668

[6] Bacci G Rocca M Salone M Balladelli A Ferrari S Palmerini E. High grade osteosarcoma of the extremities with lung metastases at presentation: treatment with neoadjuvant chemotherapy and simultaneous resection of primary and metastatic lesions. J Surg Oncol. (2008) 98:415–20. 10.1002/jso.21140 18792969

[7] Ory B Heymann M Kamijo A Gouin F Heymann D Redini F. Zoledronic acid suppresses lung metastases and prolongs overall survival of osteosarcoma-bearing mice. Cancer Interdiscip Int J Am Cancer Soc. (2005) 104:2522–9. 10.1002/cncr.21530 16270320

[8] Koshkina NV Kleinerman ES. Aerosol gemcitabine inhibits the growth of primary osteosarcoma and osteosarcoma lung metastases. Int J Cancer. (2005) 116:458–63. 10.1002/ijc.21011 15800950

[9] Sarath Chandra V Baskar G Suganthi RV Elayaraja K Ahymah Joshy MI Sofi Beaula W. Blood compatibility of iron-doped nanosize hydroxyapatite and its drug release. ACS Appl Mater Interfaces. (2012) 4:1200–10. 10.1021/am300140q 22316071

[10] Willis RE. Targeted cancer therapy: vital oncogenes and a new molecular genetic paradigm for cancer initiation progression and treatment. Int J Mol Sci. (2016) 17:E1552. 10.3390/ijms17091552 27649156 PMC5037825

[11] Shepherd JH Shepherd DV Best SM. Substituted hydroxyapatites for bone repair. J Mater Sci Mater Med. (2012) 23:2335–47. 10.1007/s10856-012-4598-2 22389101

[12] Ji Y Wang A Wu G Yin H Liu S Chen B. Synthesis of different sized and porous hydroxyapatite nanorods without organic modifiers and their 5-fluorouracil release performance. Mater Sci Eng C. (2015) 57:14–23. 10.1016/j.msec.2015.07.008 26354235

[13] Khan FU Asimullah Khan SB Kamal T Asiri AM Khan IU. Novel combination of zero-valent Cu and Ag nanoparticles @ cellulose acetate nanocomposite for the reduction of 4-nitro phenol. Int J Biol Macromol. (2017) 102:868–77. 10.1016/j.ijbiomac.2017.04.062 28428128

[14] Ahmad I Khan SB Kamal T Asiri AM Visible light activated degradation of organic pollutants using zinc–iron selenide. J Mol Liq. (2017) 229:429–35. 10.1016/j.molliq.2016.12.061

[15] Ullah I Li W Lei S Zhang Y Zhang W Farooq U Simultaneous co-substitution of Sr2+/Fe3+ in hydroxyapatite nanoparticles for potential biomedical applications. Ceram Int. (2018) 44:21338–48. 10.1016/j.ceramint.2018.08.187

[16] Wang Y Wang J Hao H Cai M Wang S Ma J. *In vitro* and *in vivo* mechanism of bone tumor inhibition by selenium-doped bone mineral nanoparticles. ACS Nano. (2016) 10:9927–37. 10.1021/acsnano.6b03835 27797178 PMC5198771

[17] Wang Y Ma J Zhou L Chen J Liu Y Qiu Z. Dual functional selenium-substituted hydroxyapatite. Interface Focus. (2012) 2:378–86. 10.1098/rsfs.2012.0002 23741613 PMC3363027

[18] Aljohani W Ullah MW Zhang X Yang G. Bioprinting and its applications in tissue engineering and regenerative medicine. Int J Biol Macromol. (2018) 107:261–75. 10.1016/j.ijbiomac.2017.08.171 28870749

[19] Fox K Tran PA Tran N. Recent advances in research applications of nanophase hydroxyapatite. Chem Phys Chem. (2012) 13:2495–506. 10.1002/cphc.201200080 22467406

[20] Gabriel LP Santos ME Jardini AL Bastos GN Dias CG Webster TJ. Bio-based polyurethane for tissue engineering applications: how hydroxyapatite nanoparticles influence the structure, thermal and biological behavior of polyurethane composites. Nanomedicine. (2017) 13:201–8. 10.1016/j.nano.2016.09.008 27720929

[21] Ma J Wang Y Zhou L Zhang S. Preparation and characterization of selenite substituted hydroxyapatite. Mater Sci Eng C. (2013) 33:440–5. 10.1016/j.msec.2012.09.011 25428093

[22] Robb CS Geldart SE Seelenbinder JA Brown PR Analysis of green tea constituents by HPLC-FTIR. J Liq Chromatogr Relat Technol. (2002) 25:787–801. 10.1081/JLC-120003036

[23] Butt MS Ahmad RS Sultan MT Qayyum MMN Naz A. Green tea and anticancer perspectives: updates from last decade. Crit Rev Food Sci Nutr. (2015) 55:792–805. 10.1080/10408398.2012.680205 24915354

[24] Zhang W Liu K Liu S Ji B Wang Y Liu Y. MicroRNA-133a functions as a tumor suppressor by targeting IGF-1R in hepatocellular carcinoma. Tumor Biol. (2015) 36:9779–88. 10.1007/s13277-015-3749-8 26156803

[25] Tsai CY Chen CY Chiou YH Shyu HW Lin KH Chou MC. Epigallocatechin-3-gallate suppresses human herpesvirus 8 replication and induces ROS leading to apoptosis and autophagy in primary effusion lymphoma cells. Int J Mol Sci. (2018) 19:E16. 10.3390/ijms19010016 29267216 PMC5795967

[26] Stadlbauer S Steinborn C Klemd A Hattori F Ohmori K Suzuki K. Impact of green tea catechin ECG and its synthesized fluorinated analogue on prostate cancer cells and stimulated immunocompetent cells. Planta Med. (2018) 84:813–19. 10.1055/s-0044-102099 29466808

[27] Tohyama Y Takano T Tanaka C He J Tohyama K Yamamura H. Induction of apoptosis by epigallocatechin-3-gallate in human lymphoblastoid B cells. Biochem Biophys Res Commun. (2007) 362:951–7. 10.1016/j.bbrc.2007.08.079 17803956

[28] Nakazato T Ito K Ikeda Y Kizaki M. Green tea component, catechin, induces apoptosis of human malignant B cells via production of reactive oxygen species. Clin Cancer Res. (2005) 11:6040–9. 10.1158/1078-0432.CCR-04-2273 16115949

[29] Yu Y Deng Y Lu BM Liu YX Li J Bao JK. Green tea catechins: a fresh flavor to anticancer therapy. Apoptosis. (2014) 19:1–18. 10.1007/s10495-013-0908-5 24081390

[30] Oh S Gwak J Park S Yang CS. Green tea polyphenol EGCG suppresses Wnt/β-catenin signaling by promoting GSK-3β- and PP2A-independent β-catenin phosphorylation/degradation. BioFactors. (2014) 40:586–95. 10.1002/biof.1185 25352148 PMC4285564

[31] Lecumberri E Dupertuis YM Miralbell R Pichard C. Green tea polyphenol epigallocatechin-3-gallate (EGCG) as adjuvant in cancer therapy. Clin Nutr. (2013) 32:894–903. 10.1016/j.clnu.2013.03.008 23582951

[32] Farhan M Khan HY Oves M Al-Harrasi A Rehmani N Arif H. Cancer therapy by catechins involves redox cycling of copper ions and generation of reactive oxygen species. Toxins. (2016) 8:37. 10.3390/toxins8020037 26861392 PMC4773790

[33] Haratifar S Meckling KA Corredig M. Antiproliferative activity of tea catechins associated with casein micelles, using HT29 colon cancer cells. J Dairy Sci. (2014) 97:672–8. 10.3168/jds.2013-7263 24359816

[34] Quesada IM Bustos M Blay M Pujadas G Ardèvol A Salvadó MJ. Dietary catechins and procyanidins modulate zinc homeostasis in. J Nutr Biochem. (2011) 22:153–63. 10.1016/j.jnutbio.2009.12.009 20471814

[35] Shan H Shi Y Quan J. Identification of green tea catechins as potent inhibitors of the polo-box domain of Polo-like kinase 1. ChemMedChem. (2015) 10:158–63. 10.1002/cmdc.201402284 25196850

[36] Jiang P Wu X Wang X Huang W Feng Q. NEAT1 upregulates EGCG-induced CTR1 to enhance cisplatin sensitivity in lung cancer cells. Oncotarget. (2016) 7:43337–51. 10.18632/oncotarget.9712 27270317 PMC5190027

[37] Fujiki H Sueoka E Rawangkan A Suganuma M. Human cancer stem cells are a target for cancer prevention using (–)- epigallocatechin gallate. J Cancer Res Clin Oncol. (2017) 143:2401–12. 10.1007/s00432-017-2515-2 28942499 PMC5693978

[38] Gratton SEA Ropp PA Pohlhaus PD Luft JC Madden VJ Napier ME. The effect of particle design on cellular internalization pathways. Proc Natl Acad Sci USA. (2008) 105:11613–18. 10.1073/pnas.0801763105 18697944 PMC2575324

[39] Yuan Y Liu C Qian J Wang J Zhang Y. Size-mediated cytotoxicity and apoptosis of hydroxyapatite nanoparticles in human hepatoma HepG2 cells. Biomaterials. (2010) 31:730–40. 10.1016/j.biomaterials.2009.09.088 19836072

[40] Tsai YJ Chen BH. Preparation of catechin extracts and nanoemulsions from green tea leaf waste and their inhibition effect on prostate cancer cell PC-3. Int J Nanomed. (2016) 11:1907–26. 10.2147/IJN.S103759 27226712 PMC4866752

[41] Kolmas J Groszyk E Piotrowska U. Nanocrystalline hydroxyapatite enriched in selenite and manganese ions: physicochemical and antibacterial properties. Nanoscale Res Lett. (2015) 10:278. 10.1186/s11671-015-0989-x 26138453 PMC4489964

[42] Ullah MW Ul-Islam M Khan S Kim Y Park JK. Structural and physico-mechanical characterization of bio-cellulose produced by a cell-free system. Carbohydr Polym. (2016) 136:908–16. 10.1016/j.carbpol.2015.10.010 26572428

[43] Tkachenko MV Kamzin AS Synthesis and properties of hybrid hydroxyapatite–ferrite (Fe3O4) particles for hyperthermia applications. Phys Solid State. (2016) 58:763–70. 10.1134/S1063783416040260

[44] Ereiba KMT Mostafa AG Gamal GA Said AH *In vitro* study of iron doped hydroxyapatite. J Biophys Chem. (2013) 04:122–30. 10.4236/jbpc.2013.44017

[45] Rossi AL Longuinho MM Tanaka MN Farina M Borojevic R Rossi AM. Intracellular pathway and subsequent transformation of hydroxyapatite nanoparticles in the SAOS-2 osteoblast cell line. J Biomed Mater Res Part A. (2018) 106:428–39. 10.1002/jbm.a.36256 29044948

[46] Zhang W Chai Y Cao N Wang Y Synthesis and characterization of selenium substituted hydroxyapatite via a hydrothermal procedure. Mater Lett. (2014) 134:123–5. 10.1016/j.matlet.2014.07.072

[47] Di Z Shi Z Ullah MW Li S Yang G. A transparent wound dressing based on bacterial cellulose whisker and poly(2-hydroxyethyl methacrylate). Int J Biol Macromol. (2017) 105(Pt 1):638–44. 10.1016/j.ijbiomac.2017.07.075 28716748

[48] Luo H Yang Y Huang F Li F Jiang Q Shi K. Selenite induces apoptosis in colorectal cancer cells via AKT-mediated inhibition of β-catenin survival axis. Cancer Lett. (2012) 315:78–85. 10.1016/j.canlet.2011.10.014 22074856

[49] Isakoff MS Bielack SS Meltzer P Gorlick R. Osteosarcoma: current treatment and a collaborative pathway to success. J Clin Oncol. (2015) 33:3029–35. 10.1200/JCO.2014.59.4895 26304877 PMC4979196

[50] Saw WS Ujihara M Chong WY Voon SH Imae T Kiew LV. Size-dependent effect of cystine/citric acid-capped confeito-like gold nanoparticles on cellular uptake and photothermal cancer therapy. Colloids Surfaces B Biointerfaces. (2018) 161:365–74. 10.1016/j.colsurfb.2017.10.064 29101882

[51] Chithrani BD Ghazani AA Chan WCW. Determining the size and shape dependence of gold nanoparticle uptake into mammalian cells. Nano Lett. (2006) 6:662–8. 10.1021/nl052396o 16608261

[52] Lu F Wu S Hung Y Mou C. Size effect on cell uptake in well-suspended, uniform mesoporous silica nanoparticles. Small. (2009) 5:1408–13. 10.1002/smll.200900005 19296554

[53] Shang L Nienhaus K Nienhaus GU. Engineered nanoparticles interacting with cells: size matters. J Nanobiotechnol. (2014) 12:1–11. 10.1186/1477-3155-12-5 24491160 PMC3922601

[54] Wang X Xu S Zhou S Xu W Leary M Choong P. Topological design and additive manufacturing of porous metals for bone scaffolds and orthopaedic implants: a review. Biomaterials. (2016) 83:127–41. 10.1016/j.biomaterials.2016.01.012 26773669

[55] Murugan R Ramakrishna S Development of nanocomposites for bone grafting. Compos Sci Technol. (2005) 65:2385–406. 10.1016/j.compscitech.2005.07.022

[56] Lowry N Brolly M Han Y McKillop S Meenan BJ Boyd AR Synthesis and characterisation of nanophase hydroxyapatite co-substituted with strontium and zinc. Ceram Int. (2018) 44:7761–70. 10.1016/j.ceramint.2018.01.206

[57] Kiełbowicz-Matuk A Rey P Rorat T. Interplay between circadian rhythm, time of the day and osmotic stress constraints in the regulation of the expression of a Solanum Double B-box gene. Ann Bot. (2014) 113:831–42. 10.1093/aob/mct303 24562097 PMC3962237

[58] Petre CE Sin S-H Dittmer DP. Functional p53 signaling in Kaposi's sarcoma-associated herpesvirus lymphomas: implications for therapy. J Virol. (2007) 81:1912–22. 10.1128/JVI.01757-06 17121789 PMC1797584

