# Supplementary material for: Catechins-Modified Selenium-Doped Hydroxyapatite Nanomaterials for Improved Osteosarcoma Therapy Through Generation of Reactive Oxygen Species
Source: Front Oncol. 2019 Jun 13;9:499. doi: 10.3389/fonc.2019.00499 (PMC6585473; doi:10.3389/fonc.2019.00499)
Supplement: Supplementary file 1 [file Data_Sheet_1.pdf]

Supplementary Materials and Methods

1. Extraction of catechins from green tea

Different catechins solutions were prepared by adding 10, 20, and 30 g green tea into 100 mL distilled water. Catechins were extracted from green tea by following a previously published protocol (Vuong et al. 2011; Lin et al. 2012; Tsai and Chen 2016) with slight modification . Briefly, the green tea leaves were grinded up to 0.8 µm to 1 mm particle size, 100 g particles were added to 500 mL of distilled water and heated at 80°C for 30 min in a temperature controlled shaking water bath. The pH was adjusted at 5.5 by using 1.0 M HCL and 1.0 M NaOH. The solution was cooled to room temperature and centrifuged at 4,000 rpm for 30 min. The collected supernatant was filtered through a 0.22 µm filter membrane and evaporated to dryness under nitrogen, followed by addition of lecithin (0.5%) and stirred. Then, Tween 80 (5%) was added and stirred again, followed by adding deionized water (94.5%). After mixing homogeneously, the mixture was sonicated for 2 h to obtain a transparent catechin emulsion with yellow appearance and processed for further experiments.

2. Stoichiometric ratios of phosphorous (P), calcium (Ca), and selenium (Se) in HAp, Se-HAp, and CC/Se-HAp nanomaterials

Table S1. Reactants molar (mMol) value and volume of deionized distilled water used for selenium-doped HA nanocomposite and HA nanoparticles.

Sample	Na_2SeO_3 (mMol)	$(NH_4)2HPO_4$ (mMol)	Ca $(NO_3)_2.4H_20$ (mMol)	CC (%)	Nominal Se/P (%)	Nominal Ca/(P+Se)
CC/Se-HAp1	0. 55	5.45	10	10	10.09	1.67
CC/Se-HAp2	0. 55	5.45	10	20	10.09	1.67
CC/Se-HAp3	0.55	5.45	10	30	10.09	1.67

Se-HAp	0.55	5.45	10	0	10.09	1.67
HAp	0	6.0	10	0	0	1.67

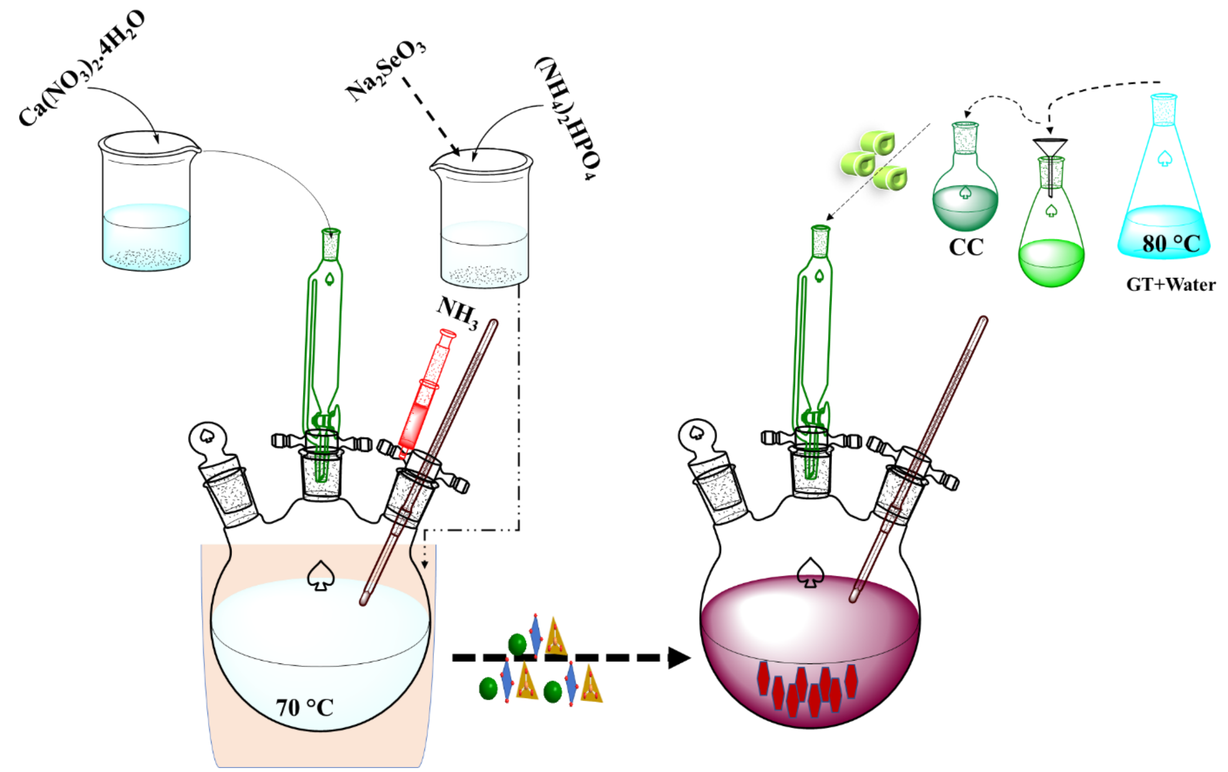

Figure S1. Illustration of preparation of HAp nanoparticles (left), selenium doping into HAp lattice (Se-HAp) (middle), and catechins modification of Se-doped HAp (CC/Se-HAp) (right).

3. Characterization of HAp nanoparticles

The synthesized pristine HAp nanoparticles, and Se-HAp and CC/Se-HAp nanocomposites were characterized for various structural and chemical properties.

3.1 XRD analysis of Se-doped HAp and CC/Se-HAp nanocomposites

The phase structure of the synthesized HAp nanoparticles, and Se-HAp and CC/Se-HAp nanocomposites were analyzed through X-ray diffraction (2θ angle from 10° to 70°) analysis with

Cu-Kα radiation (λ: 1.5418 Å), with a step size of 0.02° sec^{-1}. XRD patterns were recorded at a scanning rate of 1.2° in 2θ/min, (XRD, X'Pert PRO, PANalytical B.V., Netherlands). The crystallite size 'D' was calculated using the Deby Scherer relation (Eq. 1), while the crystallinity (X_c) was estimated using the following Eq. 2 (Erkmen, Genç, and Oktar 2007):

$$D = \left(\frac{K\lambda}{\beta_{1/2} cos\theta}\right) \qquad (1)$$

$$X_c = \left(\frac{K_A}{\beta}\right)^3 \qquad (2)$$

where 'D' (nm) represents the particle size; K = 0.94 is the Scherer constant shape factor for the HAp with hexagonal structure and '$\beta_{1/2}$' is the broadening of full width at half-maximum (FWHM) measured in radian, and 'θ' in degrees indicates the Bragg's diffraction angle, λ=1.5406Å is the wavelength of Cu-Kα radiation, 'β' is also FWHM of respective (hkl) diffraction planes, measured in degrees, and 'K_A' is a constant (K_A=0.24).

3.2 TEM analysis of Se-doped HAp and CC/Se-HAp nanocomposites

The general morphologies of the as-synthesized pristine HAp nanoparticles, and Se-HAp and CA/Se-HAp nanocomposites were examined by transmission electron microscopy (TEM, Tecnai G2 20, FEI, Holland), according to the previously reported procedure (Ma et al. 2013). Briefly, the agglomerated nanoparticles were mixed with ethanol and dispersed with ultrasonic dispersion. A couple of drops of this suspension were applied on holey carbon-coated copper grids and dried under visible light lamp. The copper grid was then observed under TEM.

3.3 SEM analysis of Se-doped HAp and CC/Se-HAp nanocomposites

The structural morphologies of platinum (Pt) sputter coated pristine HAp nanoparticles, and Se-HAp and CC/Se-HAp nanocomposites were carried out using Gemini scanning electron

microscope (SEM 300). The particle size was determined *via* software coupled with SEM and reported the mean value for each sample.

3.4 FTIR analysis of Se-doped HAp and CC/Se-HAp nanocomposites

Fourier transform infrared (FTIR) spectroscopy (Vertex 70, Bruker, German) was carried out using the classical KBr pellet system technique in transmission mode to investigate the functional groups present in the pristine HAp and confirmation of Se doping and catechins modification of HAp nanoparticles. The FTIR spectra of all samples were recorded in the spectral range of 400-4000 cm^{-1}, with 2 cm^{-1} resolution and 30 scans. For sample preparation, 3 mg of each sample was added with 200 mg KBr powders and dried at 105°C. The mixture was pulverized for a brief moment and filled into a pellet-forming die. Finally, a force of 7 tons was applied for 40 sec to obtain 13 mm transparent pellets. The FTIR spectra were recorded immediately after the background acquisition.

3.5 XRF analysis of Se-doped HAp and CC/Se-HAp nanocomposites

The atomic concentrations of different elements in the as-synthesized pristine HAp nanoparticles, and Se-HAp and CC/Se-HAp nanocomposites were determined through X-ray fluorescence (XRF) analysis (EAGLE III, EDAX Inc.,). XRF analysis was used to determine the atomic concentrations of Ca, P, and Se and their respective molar ratios such as Se/P and Ca/P in term of their respective atomic concentrations. The atomic ratio concentration of dried Se-HAp and CC/Se-HAp nanocomposites were compared against pristine HAp nanoparticles.

4. CCK-8 assay

Both types of cultured cells were trypsinized from 2D culture plate at a confluence level of 85% by using trypsin EDTA. The trypsinized cells were centrifuged at 1600 rpm for 8 min,

collected, and resuspended in respective fresh culture media and counted using a hemocytometer. Both types of cells were seeded in separate 96-well plates at a density of 1×10^4 cells/well and cultured for 48 h using the respective culture media. The CCK-8 assay kit was used to determine the cytotoxicity of hBMSCs and MNNG/HOS cell lines. Briefly, the CCK-8 reagent was diluted with MEM in 1:10 and added to each well-containing sample and incubated for 2 h at 37°C. Thereafter, 100 µl of cell suspension from each plate was transferred to a fresh 96 well plate and replicated 6 times. The CCK-8 assay was performed in triplicate for each group after the cells were cultured for the time point of 6, 12, 18, 24, 36, 42, and 48 h. At each time point, the culture media were removed, and the samples were rinsed with fresh PBS. The absorbance was measured at 450 nm using a microplate reader (Eon, BioTek, USA). The viability was determined using the following Eq.:

$$\text{Viability (\%)} = \frac{\text{Optical density of test sample}}{\text{Optical density of control}} \times 100$$

5. Primer sequences used in qPCR

Table S2. Primers used for qPCR.

S/No.	Target gene	Forward primer	Reversed primer
1	CASP3	5′-GTGCTATTGTGAGGCGGTTG-3′	5′-TCGCTTCCATGTATGATGGTT-3′
2	CASP9	5′-GTGGAAGAGCTGCAGGTGG-3′	5′-CTGCCCGCTGGATGTCCT-3′
3	p-53	5′-CGCTTCGAGATGTTCCGAGA-3′	5′-CTTCAGGTGGCTGGAGTGAG-3′
4	PTGS2	5′-TCCCTTGGGTGTCAAAGGTAAA-3′	5′-TGGCCCTCGCTTATGATCTG-3′
5	BCL-2	5′-CAGGATAACGGAGGCTGGGATG-3′	5′-TTCACTTGTGGCCCAGATAGG-3′
6	BAX	5′-AAACTGGTGCTCAAGGCCC-3′	5′-AAAGTAGGAGAGGAGGCCGT-3′
7	NF-kB1	5′-CCGGCTTCAGAATGGCAGAA-3′	5′-TATGGGCCATCTGTTGGCAG-3′
8	PTK2/FAK	5′-GGGTCCGATTGGAAACCAAC-3′	5′-CAGGATTTCTTTCCGCCCAA-3′
9	ACTIN	5′-CACCAACTGGGAGGACAT-3′	5′-GCACAGCCTGGATAGCAAC-3′

Supplementary Results

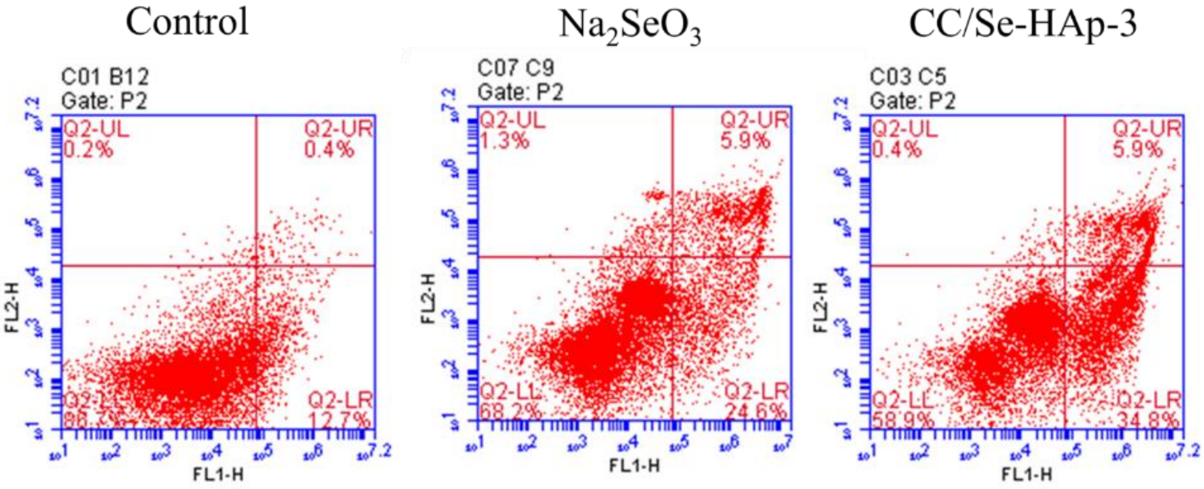

Figure S2. Quantitative analysis of cell apoptosis induced by the synthesized CC/Se-HAp in Human osteosarcoma cell line (MNNG/HOS)

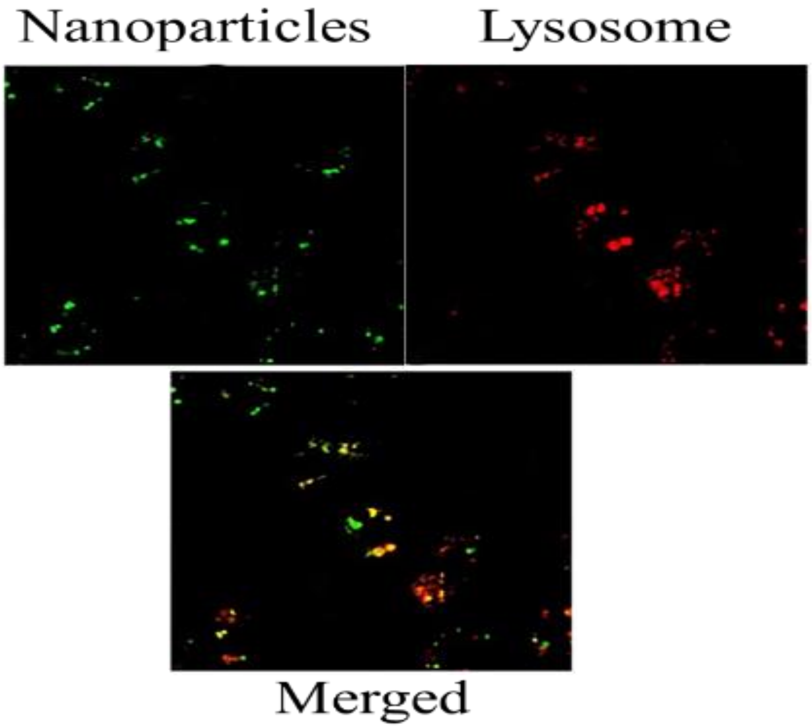

Figure S3. Detected intracellular uptake of CC/Se-HAp
